# Novel Human Tenascin-C Function-Blocking Camel Single Domain Nanobodies

**DOI:** 10.3389/fimmu.2021.635166

**Published:** 2021-03-15

**Authors:** Sayda Dhaouadi, Rahma Ben Abderrazek, Thomas Loustau, Chérine Abou-Faycal, Ayoub Ksouri, William Erne, Devadarssen Murdamoothoo, Matthias Mörgelin, Andreas Kungl, Alain Jung, Sonia Ledrappier, Zakaria Benlasfar, Sandrine Bichet, Ruth Chiquet-Ehrismann, Ismaïl Hendaoui, Gertraud Orend, Balkiss Bouhaouala-Zahar

**Affiliations:** ^1^Laboratoire des Venins et Biomolécules Thérapeutiques, Institut Pasteur de Tunis, Université Tunis El Manar, Tunis, Tunisia; ^2^Université Strasbourg, INSERM U1109 – The Tumor Microenvironment group, Hôpital Civil, Institut d'Hématologie et d'Immunologie, Fédération de Médecine Translationnelle de Strasbourg (FMTS), Strasbourg, France; ^3^Colzyx AB, Lund, Sweden; ^4^Institute of Pharmaceutical Sciences, Karl Franzens University Graz, Graz, Austria; ^5^Antagonis Biotherapeutics GmbH, Graz, Austria; ^6^Tumor Bank Centre Paul Strauss, Strasbourg, France; ^7^Friedrich Miescher Institute for Biomedical Research, Basel, Switzerland; ^8^Faculté de Médecine de Tunis, Université Tunis el Manar, Tunis, Tunisia

**Keywords:** nanobody, extracellular matrix, tenascin-c, tumor biomarker, interaction modeling, diagnostic tool, therapeutic tool, fibronectin type III repeat

## Abstract

The extracellular matrix (ECM) molecule Tenascin-C (TNC) is well-known to promote tumor progression by multiple mechanisms. However, reliable TNC detection in tissues of tumor banks remains limited. Therefore, we generated dromedary single-domain nanobodies Nb3 and Nb4 highly specific for human TNC (hTNC) and characterized the interaction with TNC by several approaches including ELISA, western blot, isothermal fluorescence titration and negative electron microscopic imaging. Our results revealed binding of both nanobodies to distinct sequences within fibronectin type III repeats of hTNC. By immunofluroescence and immunohistochemical imaging we observed that both nanobodies detected TNC expression in PFA and paraffin embedded human tissue from ulcerative colitis, solid tumors and liver metastasis. As TNC impairs cell adhesion to fibronectin we determined whether the nanobodies abolished this TNC function. Indeed, Nb3 and Nb4 restored adhesion of tumor and mesangial cells on a fibronectin/TNC substratum. We recently showed that TNC orchestrates the immune-suppressive tumor microenvironment involving chemoretention, causing tethering of CD11c+ myeloid/dendritic cells in the stroma. Here, we document that immobilization of DC2.4 dendritic cells by a CCL21 adsorbed TNC substratum was blocked by both nanobodies. Altogether, our novel TNC specific nanobodies could offer valuable tools for detection of TNC in the clinical practice and may be useful to inhibit the immune-suppressive and other functions of TNC in cancer and other diseases.

## Introduction

Tenascin-C (TNC), discovered over three decades ago, is one of the ECM molecules that is highly expressed in tumors such as breast, colorectal and gastric cancers (1–4). High TNC expression levels correlate with shortened lung metastasis-free survival in breast cancer and overall survival in glioma patients (5, 6). TNC is a large modular hexameric glycoprotein (7). Each TNC subunit displays a central oligomerization domain, followed by 14.5 epidermal growth factor (EGF)-like repeats (three disulfide bridges per EGF-repeat), 17 fibronectin type 3 (FNIII) repeats (eight constant and nine additional repeats domains that are subject to alternative splicing) and a globular fibrinogen domain (7). At physiological level, TNC is transiently expressed during organogenesis (8) and its expression is largely restricted to a few sites in the adult organism such as in some stem cell niches, tendons, and reticular fibers of lymphoid organs (9). Interestingly, high TNC levels are also found in milk of breast feeding HIV+ mothers (10). At pathological level, TNC was shown to act at multiple levels to promote tumor progression into cancer by enhancing survival, proliferation and invasion of tumor cells, driving the formation of new but poorly functional blood vessels and to corrupt anti-tumor immunity, altogether enhancing metastasis. In addition to tumors, TNC is also highly up-regulated in wound healing, fibrosis and chronic inflammation (11, 12). Recently, high TNC levels were also associated with more severe COVID19 symptoms (13). Using stochastic tumorigenesis models with engineered high and low levels of TNC it was formally proven that TNC indeed is a promoter of tumor progression (14). TNC is inducing and activating a wide range of cellular signaling pathways such as Wnt, Notch, JNK and TGFβ (14–17). TNC also acts on stromal and immune cells thereby promoting tumor angiogenesis and immune escape (18–22). The distinct spatio-temporal expression pattern of TNC is highly regulated (23). *In vitro* studies demonstrated that various stimuli such as EGF, TGFβ, b-FGF, and TNF-α, can induce expression of TNC in breast cancer stroma (5, 24). In cancerous breast tissues, EGF induced TNC via its receptor EGFR which activated oncogenic Ras signaling. Mammary tumor cells also produced transforming growth factor β1 (TGFβ1), which induced TNC expression in the surrounding stroma (25, 26). Due to defective autophagy TNC seems to be highly abundant in triple negative breast cancer (27). An overview of factors regulating TNC expression is presented in Giblin et al. (23).

Given its high expression in cancer tissues as well as its inflammation promoting actions, several efforts have been launched to specifically detect TNC *in situ* as well as to inhibit its main pathological effects. These approaches included down regulation of TNC expression with siRNA or aptamers (28–32) and the use of TNC-specific antibodies for the delivery of drugs or radiotherapy (33–35). Moreover, numerous monoclonal antibodies recognizing TNC have been developed. However, all generated tools have their intrinsic limitations and caveats. In particular, antibodies may not reach the target tissue or can raise an immune response and formalin fixation, usually used in routine pathology service, can impair epitope recognition (36, 37). Thus, better molecular tools are needed for specific and sensitive recognition and potential targeting of TNC. To overcome these limitations, recombinant nanobodies (Nbs) with their remarkable characteristics (i.e., high stability, solubility and specificity and low immunogenicity) may provide a solution (38–43).

Here, we have generated two “best in class” nanobodies (Nb3 and Nb4) that recognize specifically TNC with high affinity by ELISA and staining of formalin fixed and fresh frozen tissues, emphasizing novel opportunities for early diagnosis and potential monitoring of cancer progression. Therefore, these nanobodies may be useful for applications in routine cancer diagnosis and for future *in vivo* targeting of TNC in cancer. On the other hand, as TNC impairs cell adhesion on a fibronectin substratum, we determined whether the nanobodies abolished this function of TNC. Indeed Nb3 and Nb4 restored adhesion of human osteosarcoma and mesangial cells on the fibronectin/TNC substratum. Interestingly, we observed that immobilization of DC2.4 dendritic cells on a TNC substratum in context of CCL21 (22) was blocked by Nb3 and Nb4. Finally, by modeling the Nb/TNC interaction we determined the putative amino acid residues involved in complex formation. Altogether, we demonstrated that our two novel TNC-specific nanobodies display valuable characteristics for detection of TNC *in situ*, and revealed their potential as therapeutic tools for inhibition of immune-suppressive and other functions of TNC.

## Materials and Methods

### Purification of Recombinantly Expressed hTNC

HEK 293/hTNC cells, previously stably transfected with the human TNC coding sequence (hTNC) were used to produce hTNC as previously described (44, 45). Briefly, cells were cultured in Dulbecco's Minimal Essential Medium (DMEM, catalog number 11995040 Gibco Life Technologies, Inc., Paisley, Scotland) supplemented with 10 % (v/v) fetal calf serum (FCS, catalog number 2-01F90-I BioConcept, Allschwil, Swizerland), 10.25 μg/mL G418 and 1.5 μg/mL puromycin under a 5% CO_2_ atmosphere at 37°C. The recombinant hTNC was purified from the conditioned medium lacking FCS as previously described (44). Briefly, fibronectin was removed from the conditioned medium by gelatin-agarose affinity chromatography (46, 47), and the flow through was purified by a nickel affinity chromatography column (48). The purity of the protein was checked by Coomassie Blue stained 7% SDS-PAGE and by western-blot, under reducing and non-reducing conditions. The concentration of hTNC was determined by Bradford assay (catalog number 500-0006 Bio-Rad Laboratories, Hercules, CA, USA).

### *E. coli* Strains and Vector

The phage display vector pMECS of 4,510 bp was utilized to construct the VHH library, hosted in *E. coli* strain TG1 (generously provided by Prof. Serge Muyldermans, VUB Brussel, Belgium). This phagemid vector contains a sequence encoding a PelB leader signal to secrete the cloned VHH-encoded Nb in the periplasm with two C-terminal Hemagglutinin (HA) and 6X Histidine (6X His) tags for VHH-detection, when hosted in *E. coli* strain WK6 (49).

### Generation of the Phage-Display VHH-Library

The anti-hTNC nanobody phage-display VHH-library was constructed as previously described with slight modifications (39, 49, 50). Briefly, 3 days after the last boost of antigen injection, 150 mL of anti-coagulated blood sample was collected from the jugular vein of the immunized dromedary as recently detailed (51). Peripheral blood mononuclear cells (PBMCs) were extracted by density gradient centrifugation using Lymphoprep (catalog number 17-829 LONZA, Basel, Switzerland). Subsequently, total RNA was extracted and purified. An amount of 40 μg of total RNA was reverse transcribed into cDNA with oligo-dT primer and the SuperScript II First-Strand Synthesis System for RT-PCR (catalog number 18064-014 Invitrogen, Carsbad, CA, USA). Thereafter, cDNA fragments were used as template to amplify heavy-chain IgG encoding variable domains using specific primers [CALL001 (5′-GTCCTGGCTGCTCTTCTACAAGG-3′) and CALL002 (5′-GGTACGTGCTGTTGAACTGTTCC-3′)]. The 700 bp PCR fragment (VHH-CH2 without CH1 exon, corresponding to heavy-chain antibodies) was purified from a 1% agarose gel using the Qiaquick gel extraction kit (catalog number 28704 Qiagen, Hilden, Germany). Subsequently, these sequences were used as template in a nested PCR to amplify VHH-only variable domains with nested-PCR primers [SM017 (5′-CCAGCCGGCCATGGCTGCATGGTGCAGCTGGTGGAGTCTGG-3′) and PMCF (5′-CTAGTGCGGCCGCTGAGGAGACGGTGACCTGGGT-3′)], annealing at the Framework 1 and Framework 4 regions, including NcoI and NotI restriction sites, respectively (catalog numbers R0193T and R3189M New England Biolabs, UK, respectively). The PCR product was ligated into the pMECS phagemid vector (T4 DNA Ligase, catalog number 15224-041 Invitrogen, Carsbad, CA, USA) using a molar ratio 1:3 in favor of the inserts. Freshly prepared electro-competent *E. coli* TG1 cells were transformed by the ligated product and plated overnight (O/N) on selective Luria-Bertani Miller (LB) media supplemented with (100 μg/mL) ampicillin (catalog number 271896 Sigma Aldrich, MO, USA) and glucose 2% (catalog number G8270 Sigma Aldrich, MO, USA). Colonies were recovered from the overnight-incubated plates at 37°C. Library size was estimated by serial dilutions.

### Selection of Anti-hTNC Nanobodies (Nbs)

A representative repertoire of the VHH library was displayed on phage particles using M13KO7 helper phage infection (catalog number 170-3578 New England, BioLabs, UK). Three consecutive rounds of immuno-affinity selection were carried out on 96-well microtiter plates (catalog number M5785-1CS Sigma Aldrich, MO, USA) pre-coated with hTNC (1 μg/panning, O/N at 4°C). After each round of biopanning, bound phage particles were eluted (100 mM triethylamine, pH 10.0, catalog number T0886 Sigma Aldrich, MO, USA) and immediately neutralized with 1 M Tris-HCl, pH 7.4 (catalog number CE234 GeneON, Germany) and used to infect exponentially growing TG1 *E. coli*. Following the third round of biopanning, individual colonies were randomly picked. VHH expression was induced with 1 mM isopropyl-D-thiogalactopyranoside (IPTG, catalog number 2900245 5PRIME, Germany) in the periplasmic bacterial compartment. Solid phase ELISA of each periplasmic extract was carried out on hTNC (1 μg/mL), using a mouse anti-HA antibody (catalog number H9658 Sigma Aldrich, MO, USA) and goat anti-mouse IgG-peroxidase antibody (catalog number A9044 Sigma Aldrich, MO, USA).

### VHH Sequence Analysis

The VHH sequences of clones that scored positive in periplasmic extract-ELISA were determined using the Genomic platform of Institut Pasteur de Tunis facilities (ABI Prism 3100 genetic analyzer; Applied Biosystems, Foster City, CA, USA). The VHH nucleotide sequences were obtained using the ABI PRISM^TM^ BigDye Terminator v3.1 Cycle Sequencing Reaction Kit (catalog number 4337454 Applied Biosystems, USA).

### Production, Purification, and Characterization of hTNC-Specific Nbs

Recombinant vectors of selected positive clones with highest binding capacity to hTNC were used to transform WK6 electrocompetent cells. Nb production was performed in shake flasks by growing each recombinant bacteria in Terrific Broth medium (TB, catalog number 743-29175 BD Biosciences, FL, USA) supplemented with ampicillin (100 μg/mL) and 0.1% glucose. The Nb periplasmic expression was subsequently induced with 1 mM IPTG, O/N at 28°C. The periplasmic extract obtained by osmotic shock was loaded on a His-Select column (NiNTA, catalog number 1018544 Qiagen, Hilden, Germany). The His-tagged hTNC-specific Nbs were eluted with 500 mM imidazole (catalog number I-0125 Sigma Aldrich, MO, USA) and an amount of 5 μg was checked on a 15% SDS gel upon-PAGE (Bio Rad), following dialysis towards PBS with a 12 kDa cut-off membrane (catalog number D9527-100FT Sigma Aldrich, MO, USA). The final yield was determined using Bradford assay (catalog number 500-0006 Bio-Rad Laboratories, Hercules, CA, USA) and the molar concentration was estimated using the theoretical extinction coefficient of the VHH sequence. The specificity of the purified anti-hTNC nanobodies was assessed by ELISA. Briefly, 0.5 μg/mL of hTNC was coated onto microtiter plates O/N at 4°C and unspecific sites were blocked with 1% (w/v) gelatin (catalog number 48723 Fluka Analytical, USA) supplemented with PBS/0.05% Tween-20 at 37°C for 2 h. Affinity-purified Nb was added (5 μg/mL, 1 h). Following a washing step, bound Nb was detected with a mouse anti-HA antibody (catalog number H9658 Sigma Aldrich, MO, USA) and revealed with a goat anti-mouse IgG-peroxydase conjugate (catalog number A9044 Sigma Aldrich, MO, USA).

### Assessement of Nb Binding Affinity

The assessement of Nb binding affinity was performed using two methods: (i) indirect ELISA was carried out using a serial Nb dilution ranging from 5 × 10^−7^ to 5 × 10^−12^ M, as described above; (ii) Isothermal Fluorescence Titration (IFT) was performed using recombinant murine TNC (mTNC, 700 nM, 0.01% Tween-20), as previously described with slight modifications (52). Briefly, the Nb concentration varied from 500 to 3,500 nM. The fluorescence emission spectra for mTNC/Nb complexes were collected and subsequently subtracted from emission spectra for mTNC and the resulting curves were then integrated. The mean values resulting from three independent measurements were plotted against the concentration of the added Nb. The resulting binding isotherms were analyzed by nonlinear regression using the program Origin (Microcal Inc., Northampton, MA, USA). The following equation describes the bimolecular association reaction, where Fi is the initial and Fmax is the maximum fluorescence values. The KD is the dissociation constant, and [mTNC] and [Nb] are the total concentrations of the mTNC and the Nb ligand, respectively:

$$\begin{array}{l} \text{F}={\text{F}}_{\text{i}+}{\text{F}}_{\text{max}}[\text{KD}+[\text{mTNC}]+[\text{Nb}]\\ \;-\frac{\sqrt{{\left(\text{KD}+\left[\text{mTNC}\right]+\;\left[\text{Nb}\right]\right)}^{2}\;-4\left[\text{mTNC}\right][\text{Nb}]}}{2[\text{mTNC}]}] \end{array}$$

### Negative Electron Microscopy Imaging

The Nb/ hTNC interaction complexes were visualized by negative staining and electron microscopy as previously described (53). Each Nb (20 nM) was conjugated with 5 nm colloidal gold particles (AuNPs) according to routinely used procedures (54). AuNP-Nb conjugates were incubated with hTNC (20 nM) for 30 minutes (min) at room temperature (RT) and subsequently negatively stained with 2% uranyl acetate. Specimens were assessed and electron micrographs were taken at 60 kV with a Phillips EM-410 electron microscope using imaging plates (Ditabis).

### Western Blot Analysis of TNC Specific Nb

Cell lysates (40 μg) from HEK293, HEK293/hTNC, (20 μg) NT193 and subclone NT193-1 cells (generated by limited dilution), RAW267 macrophages (ATCC) and DC2.4 dendritic cells (22) in RIPA buffer (catalog number R0278 SIGMA Aldrich, MO, USA) or purified human hTNC (hTNC, 100 ng) and murine TNC (mTNC, 50 ng) were boiled at 100°C for 5 min, before loading on a 4–20% gradient SDS/PAGE gel (catalog number 456-8095 Mini-PROTEAN TGX^TM^, Bio-Rad Laboratories, Hercules, CA, USA), then, transferred onto a PVDF membrane (catalog number 1620174 Bio-Rad Laboratories, Hercules, CA, USA). After blocking with 5% milk, PBS/0.1% Tween-20 (catalog number 1706404 Bio-Rad Laboratories, Hercules, CA, USA) the membrane was incubated O/N at 4°C with Nb3 or Nb4 (2 μg/mL). After three washing steps, the membrane was first incubated with a mouse anti-HA antibody (1 h 30 min at RT), and then with the anti-mouse IgG horseradish peroxidase conjugate diluted at 1:1,000 (catalog number AB_772209, NXA931, Amersham GE Helthcare, USA). Immunocomplexes were revealed with ECL (catalog number 28 980926 Amersham GE healthcare, USA). A prestained protein ladder (10–250 kDa, catalog number 06P-0211 Euromedex, France) was used. Mouse monoclonal antibody B28.13 (1 μg/mL), raised against hTNC was used as a positive control (55).

### Immunofluroescence Assay

Glioblastoma cell xenografts had previously been generated by subcutaneous injection of 2 × 10^6^ U87MG or U87MG-shTNC (TNC knockdown) cells into the flank of a nude mouse (56). Frozen (−80°C) sections were cut (7 μm thickness), fixed with 4% paraformaldehyde (PFA) (catalog number 30525-89-4 Sigma Aldrich, MO, USA) for 15 min at RT, and permeabilized with 0.5% Triton X-100 in PBS and blocked with 10% normal donkey serum (NDS) in PBS for 2 h at RT (catalog number 017-000-121 Jackson ImmunoResearch Inc, USA). Sections were co-stained with the Nb and B28.13 antibody diluted in PBS, 10% NDS O/N at 4°C, rabbit anti-HA antibody (ab236632 abcam, UK, 1:1,000 dilution, 90 min at RT) and, donkey anti-mouse antibody labeled with Texas Red fluorophore (catalog number PA1-28626 Invitrogen, Carsbad, CA, USA), and donkey anti-rabbit antibody labeled with Alexa Fluor 488 Green fluorophore (catalog number AB-2313584 Jackson Immuno Research Inc, USA) were used (1:1,000 dilution for 90 min at RT). After each antibody incubation, sections were washed five times with PBS. For staining of cell nuclei, sections were incubated with 4', 6-diamidino-2-phenylindole (DAPI, 0.2 μg/mL, catalog number 32670 Sigma Aldrich, MO, USA) for 10 min at RT. Slides were sealed with a polymerization medium (Fluorsave^TM^ Reagent, Calbiochem) underneath the coverslips and stored at 4°C until analysis. Pictures were taken with an AxioCam MRm (Zeiss) camera and Axiovision software.

### Human Tumor Samples and Analysis by Immunohistochemistry

Surgically removed tongue tumors, Formalin-Fixed Paraffin-Embedded (FFPE) embedded in FFPE, were retrieved from the tumor bank of the Centre Paul Strauss (Strasbourg, France). The FFPE-embedded 17/18G percutaneous needle biopsy of an hepatic metastasis derived from carcinoma of the gall bladder (CGB) was collected as part of a study involving human participants approved by the Mongi Slim University Hospital (MSUH) Committee on Medical Ethics (La Marsa, Tunisia) and the Ethikkommission Nordwest-und Zentralschweiz (Switzerland). Informed consent was obtained for all subjects. Characteristics of patients with oral squamous cell carcinoma (OSCC), or CGB liver metastasis, are summarized in Supplementary Table 1.

Immunohistochemical staining of OSCC samples was performed on serial 5 μm deparaffinized tumor sections. For hTNC staining, intrinsic peroxidase was blocked by incubating sections with 3% hydrogen peroxide for 15 min and antigen retrieval was performed in Sodium Citrate (10 mM) buffer pH 6.0 at 95°C. Sections were blocked in 5% goat serum for 1 h, then incubated ON/4°C with rabbit anti-TNC antibody (#19011, Millipore, 1 μg/mL) or anti-hTNC Nb (2 μg/mL). After PBS rinsing, sections were incubated with biotinylated goat anti-rabbit or goat anti-lama antibodies (1 h at RT) then avidin-biotin (PK-4000, VECTASTAIN ABC Kit, Vector Lab, California, USA). Staining was revealed with 3, 3 ′-Diaminobenzidine developing solution (SK-4100, DAB, Vector Lab, California, USA) then sections were counterstained with hematoxylin. After embedding in aqueous mounting medium, sections were examined using a Zeiss Axio Imager Z2 microscope. Pictures were taken with an AxioCam MRm (Zeiss, Axiovision) camera. The image acquisition setting (microscope, magnification, light intensity, exposure time) was kept constant per experiment and in between conditions. The origin of the tumor sample, patient gender, TNM stage, presence of metastasis and sampling date are depicted in Supplementary Table 1.

Immunohistochemical stainings of the CGB liver metastasis sample were performed on a Ventana Discovery Ultra instrument (Roche Diagnostics). The procedure RUO Discovery Universal was used with 40 min CC1 pre-treatment and anti-TNC B28.13 (1:5,000), Nb3 (1:100) or Nb4 (1:50) were applied manually and incubated for 1 h at 37°C. For Nb3 and Nb4, a rabbit anti-HA antibody (C29F4, Cell Signaling), used as a linker to detect the nanobodies, was applied manually (1:200) and incubated for 1 h at 37°C. Then, an anti-mouse antibody used for B28.13 (ImmPRESS reagent kit peroxidase anti-mouse Ig MP-7402, Vector Laboratories) or an anti-rabbit antibody used for Nb3 and Nb4 (ImmPRESS reagent kit peroxidase anti-rabbit Ig MP-7401, Vector Laboratories) were applied manually (200 μl) and incubated for 32 min at 37°C. Finally, the ChromoMap DAB kit (Roche Diagnostics) was used for detection and slides were counterstained with Hematoxylin II and Bluing Reagent (Roche Diagnostics) for 8 min.

### Boyden Chamber Transwell Chemoretention Assay

Boyden chamber transwell chemoretention assay of DC2.4 dendritic cells toward CCL21 was carried out as described previously (22). The bottom of the chamber was filled with DMEM containing human CCL21 (200 ng/mL, catalog number 366-6C-025 R&D Systems, Minneappolis, USA). The lower surface of the transwells was coated with purified horse fibronectin (FN) (37), rat collagen type I (Col I, catalog number 354236 BD Biosciences, FL, USA) or hTNC at a final concentration of 1 μg/cm^2^ and incubated O/N with blocking solution alone or with the Nb, respectively. DC2.4 (5 × 10^5^) cells resuspended in 150 μL of 1% FBS-complemented DMEM were placed into the top chamber of the transwell system. After 5 h of incubation at 37°C in 5% CO_2_, cells on the lower side of the insert were fixed with PFA and stained with DAPI before cell counting.

### Adhesion Assay

The adhesion assay was carried out on human KRIB osteosarcoma and human MES mesangial cells. Precisely, 96-well plates were coated with 1 μg/cm^2^ FN, hTNC or with FN and hTNC together. Nbs were added at 500 nM after the coating for 1 h at 37°C. Plates were rinsed with PBS and the non-coated plastic surface was blocked with 1% BSA for 1 h. After blocking, KRIB osteosarcoma and MES were plated for 3 and 2 h, respectively, at 37°C in a humidified atmosphere with 5% CO_2_. After incubation, non adherent cells were removed by PBS washing and spread cells were stained with cristal violet and counted.

### Statistical Analysis

Statistical differences were analyzed by a two-way ANOVA test or a Kruskal-Wallis t-test, Student's test and Dunn's post-test. Statistical analyses were performed using the GraphPad Prism software. *p*-values < 0.05 or < 0.005 were considered as statistically significant. Data are expressed as the mean ± SEM.

### Structural Modeling of the Nb – TNC Interaction

For the Nb - TNC structral interaction modeling the Rosetta Antibody application (57, 58) from “ROSETTA 3.8”^1^ was used. Selection of the top 10 Model was done according to Rosetta scoring based on system energy. Structural information about the 5th TNC fibronectin type III repeat (TN5) was extracted from the protein data bank deposited under the PDB code: 1TEN. The TN5 structure was refined using the ROSETTA relax application which then was used to map potential interaction sites in the selected Nb through the ZDOCK docking tool, version 3.0.2 (59). Structure and complex interactions were then visualized via the molecular visualization PyMOL software (60).

## Results

### Generation of an Immune VHH Library to Produce hTNC- Specific Nanobodies

An immune response against hTNC had been elicited in the immunized dromedary as previously described (51). From PBMC, total RNA was extracted and cDNA was prepared. This cDNA was used as template to perform the first PCR using primers CALL001 and CALL002 specific for the variable domains of the heavy-chain isotypes (IgG1, IgG2, and IgG3), subsequently leading to the co-amplification of VH and VHH coding domains (Supplementary Figure 1A). As expected, because of the presence of CH1 domain in conventional antibodies (IgG1) and different hinge size in non-conventional antibodies (HCAbs), three PCR products were observed by agarose gel electrophoresis: the 900, 790, and 720 bp fragments corresponding to the VH-CH1-Hinge-CH2 of the IgG1 and the VHH-Hinge-CH2 exons of the IgG2 and IgG3, respectively (Supplementary Figure 1A). Selective only-VHH fragments were successfully amplified using nested PCR specific-primers and cloned in pMECS phagemid (see above). The obtained hTNC-specific VHH library was estimated to contain approximately 3 × 10^6^ CFU/mL independent clones. The insert size of 19 randomly chosen clones was investigated by PCR. The library insertion rate with a VHH insert of the expected size was 78.94% (Supplementary Figure 1D).

**Figure 1:**
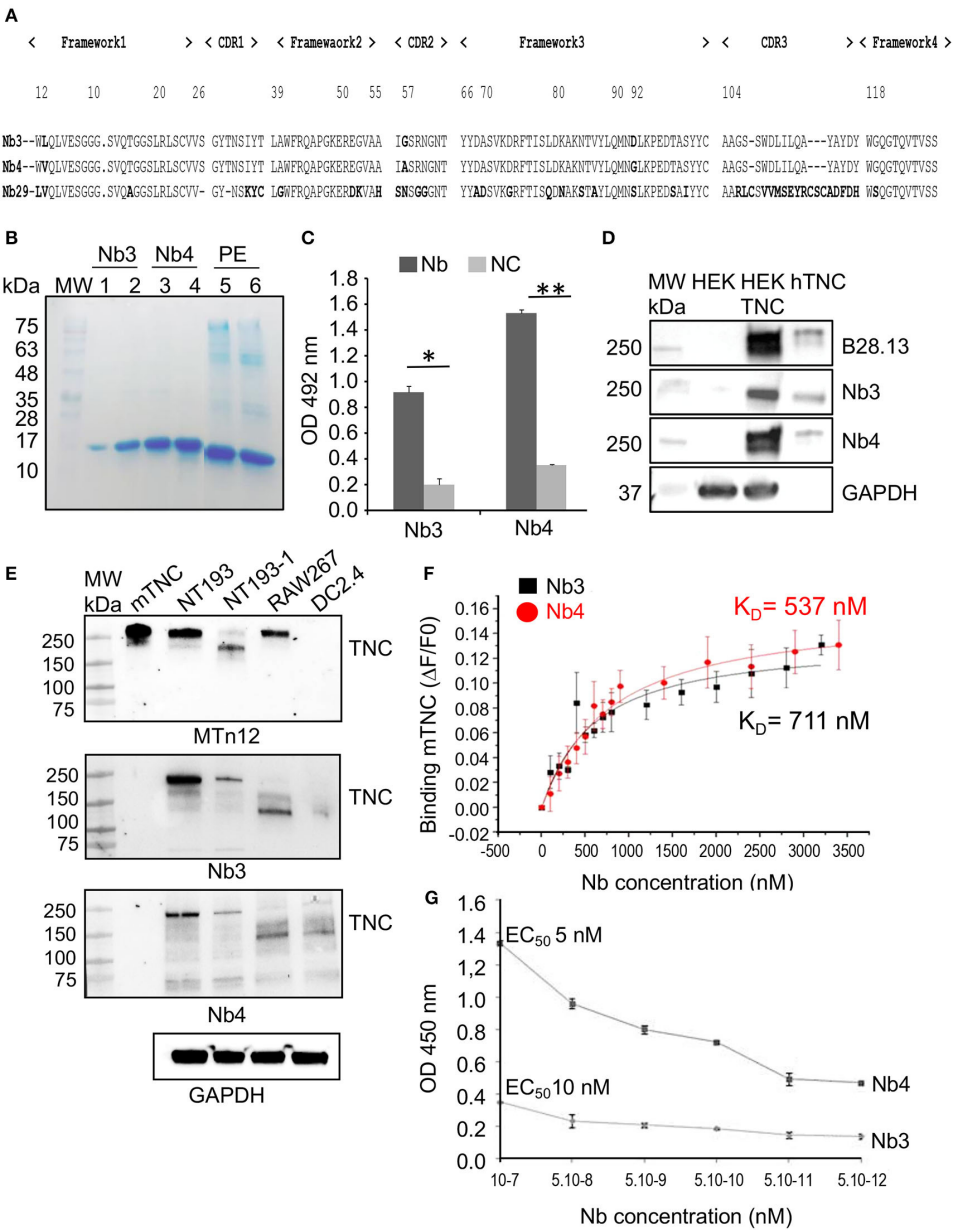
Specificity of the purified nanobodies for hTNC **(A)** Comparative alignment of amino acid sequences of nanobodies Nb3, Nb4, and Nb29 showing four amino acid hallmark changes at positions F42, E49, R50, and G52 according to the IMGT Scientific chart analysis for the V-Domain. Positioning of CDRs1-3 and Frameworks 1–4 are indicated. **(B)** SDS-PAGE analysis of the purified nanobodies Nb3 and Nb4. The nanobodies were expressed in bacteria and purified bacterial lysate was separated on a 15 % SDS-PAGE gel that was stained with Coomassie blue. Lanes represent MW: Prestained molecular weight marker, size indicated in kDa. 1, 2: Nb3 eluates 1 and 2. 3, 4: Nb4 eluates 1 and 2. 5: Purified periplasmic extract (PE) from Nb3 after induction. 6: Purified periplasmic extract from Nb4 after induction. Nb3 and Nb4 are visible at 15 kDa, the respective molecular weight of a nanobody. **(C)** Binding specificity assessement of Nb3 and Nb4 An amount of 50 ng hTNC was coated onto microtiter plates, and 500 ng (100 μl) nanobodies were added. After incubation with a mouse anti-HA antibody and then anti-mouse HRP, absorbance at 492 nm was measured by an ELISA reader. NC, no coating. Values were the means of 3 independent experiments. Mean ± SEM, *p < 0.05, **p < 0.01, Student's t-test. **(D)** Western blot analysis of Nb3 and Nb4 An amount of 40 μg of total cell lysate from parental HEK293 (HEK) (devoid of TNC) and HEK:TNC (engineered to express hTNC) and 100 ng of purified hTNC were analyzed by Western blot for detection of TNC by B28.13 (monoclonal anti-hTNC antibody) or Nb3 and Nb4 (2 μg/mL). GAPDH was used as loading control. Representative result, *n* = 3. **(E)** Western blot of 50 ng of purified mTNC (22) and 20 μg of total cell lysate of the indicated murine cells with GAPDH as loading control. Detection of TNC with the MTn12 antibody or Nb3 and Nb4, respectively. Representative result, *n* = 2. **(F)** Determination of the effective concentration (EC50) at which 50% of epitopes in hTNC are occupied by Nb3 (*diamonds*) and Nb4 (*squares*), respectively. The experiment was done in triplicates. Mean ± SEM. *p < 0.05, Two-way ANOVA test. **(G)** Binding affinities of Nb3 and Nb4 for recombinant mTNC as measured by isothermal fluorescence titration. The experiment was done three times.

A representative aliquot of the TG1 cells harboring the VHH antibody repertoire was rescued with the M13KO7 helper phage to produce phage particles expressing the nanobodies. Following three rounds of phage display selection on solid-phase coated hTNC, enrichment for hTNC-specific phage particles was observed from the first round of panning onwards (Supplementary Table 2). Twenty-five periplasmic extracts selected from randomly picked individual clones of biopanning rounds were checked by ELISA. Only clones scoring positively by ELISA without binding to unspecific proteins (AahI and BotI toxins, respectively) were retained (Supplementary Figure 1E). In total, eight recombinant clones displaying highest recognition and binding to the hTNC from the sequenced VHHs were selected. Two recombinant clones displayed identical amino-acid sequences (Nb3 and Nb5). The Nb3, Nb4, and Nb29 were selected for further investigation. As illustrated in Figure 1A, all hTNC-specific Nb sequences (Nb3, Nb4, and Nb29) exhibit the VHH hallmark amino acid residues in the framework-2 region (FR2 Phe42, Glu49, Arg50, and Gly52) and the conserved Trp at position 118 of the anchoring region. According to their common CDR1 sequence, Nb3 and Nb4 are most likely derived from the same V-D-J rearrangement and share the same B-cell progenitor (40). The difference in CDR3 length of the Nb29 corresponding sequence is remarkable (21 amino-acid residues). Interestingly, the Nb29 has a total of six cysteins (at positions 23, 33, 103, 108, 117, and 119) and therefore harbors two additional interloop disulfide bonds, in addition to the FR1/FR3 conventional ones (Cys23/Cys103). Nevertheless, because of its moderate binding to hTNC and low yield, Nb29 was not further investigated here.

The CDR3 residue length within the Nb3 and Nb4 sequences (17 amino-acid residues) was identical and no difference was noticed. The only divergence in the Nb3 and Nb4 sequences was observed at position 2 (Leu substituted by Val in FR1), at position 52 (Gly substituted by Ala in CDR2) and at position 92 (Asp substituted by Gly in FR3), respectively, suggesting an interaction with a common epitope on TNC.

### Production and Purification of hTNC Specific Nanobodies

Only clones that scored highly positive (Supplementary Figure 1E) were further used for flask production and Immobilized Metal Affinity Chromatography purification (IMAC), according to Hmila et al. (61). Briefly, production of each soluble anti-hTNC Nb was accomplished by transformation of *E. coli* WK6 cells with the corresponding recombinant phagemid. The amber stop codon located between the VHH insert and the gene III within the pMECS phagemid, resulted in expression of the Nb as soluble protein in the periplasm compartment of *E. coli*, leading to rapid IMAC purification of the Nb. As expected, the Coomassie-stained SDS/PAGE gel revealed the apparent molecular weight of 14 kDa (Figure 1B). The Nb3 and Nb4 production yields were estimated ranging from 0.6 to 0.8 mg/L, respectively, when flask cultured in TB medium. No bands indicative of contaminants or Nb degradation were detected.

Nb4 (5 μg/mL) displayed a higher ELISA binding titer toward hTNC (0.5 μg/mL, OD_492nm_ = 1.526), compared to Nb3 (5 μg/mL, OD_492nm_ = 0.913) and to the irrelevant nanobody (anti-BotI toxin nanobody, 5 μg/mL, OD_492nm_ = 0,118) (Figure 1C, Supplementary Figure 2). Furthermore, immunoblotting assays revealed that both nanobodies showed a specific recognition of not only the purified hTNC (100 ng) but also of TNC in the supernatant from HEK293/TNC cells. Parental HEK293 cells did not express TNC and also showed no signal with Nb3 nor Nb4 (Figure 1D).

As human and murine TNC are highly conserved (62) we used Nb3 and Nb4 for detection of murine TNC by western blot. Whereas, the monoclonal anti-TNC antibody MTn12 recognized recombinant mTNC expressed in HEK293/TNC cells indicated by the appearance of multiple bands, Nb3 and Nb4 did not recognize these TNC species (Figure 1E). There were several higher molecular weight bands of TNC recognized by MTn12 in recombinant TNC, NT193, and RAW267 cells. However, one or more of these bands at 250 kDa were well-recognized by Nb3 and Nb4 in NT193 cells but poorly in HEK293 and RAW267 cells. On the contrary TNC proteoforms (between 150 and 250 kDa) that were recognized by Nb3 and Nb4 in NT193-1, RAW267 and DC2.4 cells were not recognized by MTn12 (Figure 1E). There are several explanations for this result. First it is conceivable that the epitope recognized by MTn12 (which is not known) is different to that recognized by Nb3 and Nb4. Second Nb3 and Nb4 recognize a particular TNC conformation as epitope which is lost upon denaturation by SDS and boiling. Third, glycosylation may have an impact on the conformation of the epitope recognized by Nb3 and Nb4. Recently it was shown that N-glycosylation in TNC (in particular within TN5) impacted binding of the envelope protein of HIV (10). Apparently the conformation of the epitope recognized by Nb3 and Nb4 is still available in NT193 and the other murine cells despite denaturation as seen in Figure 1E. These bands are specific for TNC since unspecific anti-HA bands are below 85 kDa (63). In conclusion, Nb3 and Nb4 may recognize a conformational epitope in TNC that could be sensitive to denaturation and/or N-glycosylation which has to be further investigated in the future.

### Assessment of Nb3 and Nb4 Affinities for TNC by Isothermal Fluorescence Titration

By Isothermal Fluorescence Titration (IFT), we investigated the binding of Nb3 or Nb4 to fluorescently tagged mTNC until signal saturation and determined the dissociation constant (K_D_) as 711 × 10^−9^ M (Nb3) and 537 × 10^−9^ M (Nb4) that indicates a robust interaction (Figure 1F). In addition, ELISA assays were performed with Nb molar concentrations ranging from 5 × 10^−7^ to 5 × 10^−12^ M and revealed specific binding with a 50% Effective Concentration (EC_50_) of both Nb3 and Nb4 binding to hTNC at 10 and 5 nM, respectively, again revealing a strong interaction (Figure 1G).

### Detection of the Nanobody Binding Site in TNC by Negative Electron Microscopy

**Figure 2:**
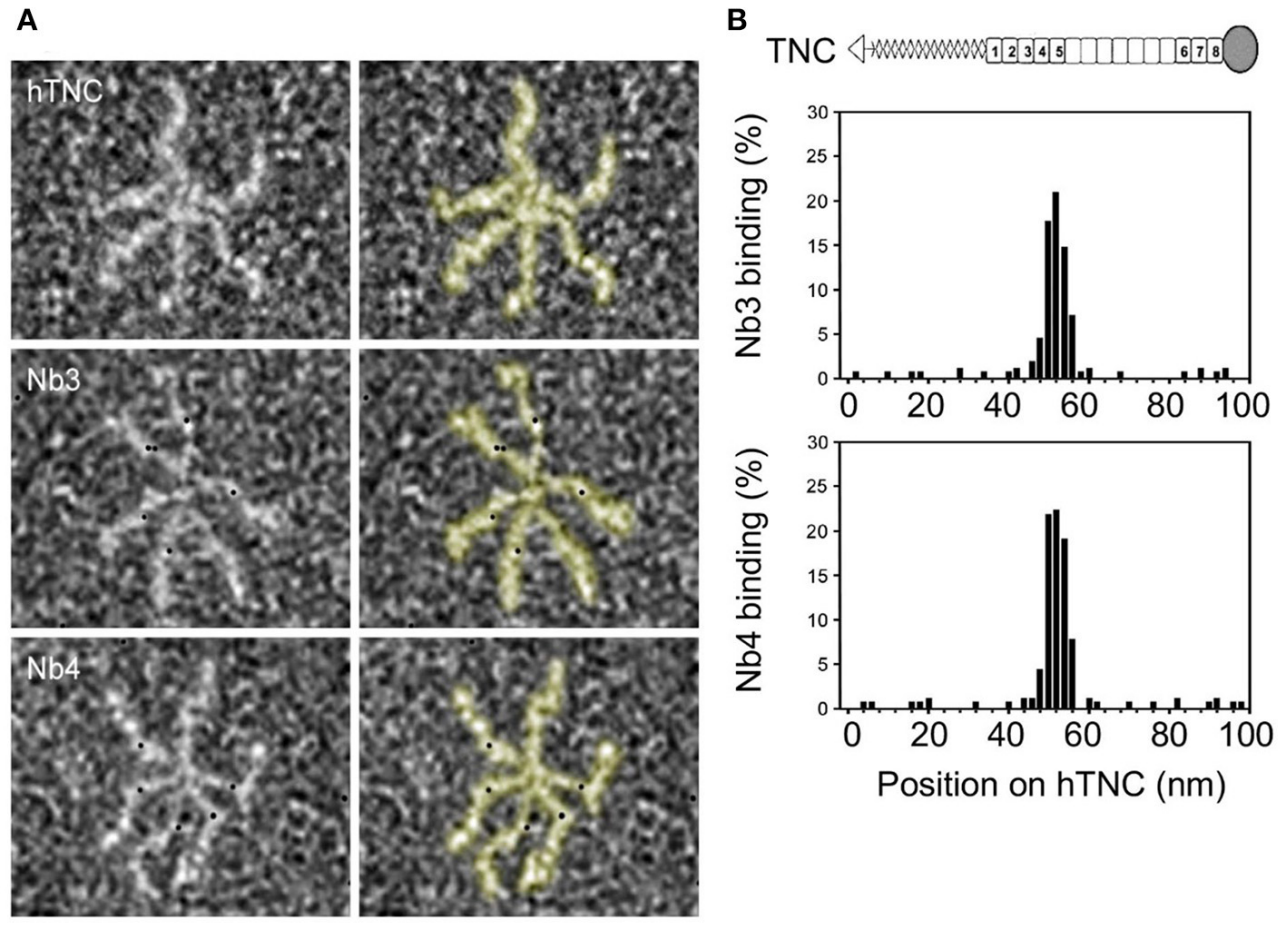
Identification of interaction sites of Nb3 and Nb4 in hTNC. **(A)** Binding of gold-labeled Nb3 and Nb4 to hTNC was determined by negative staining and transmission electron microscopy. The hTNC molecule in the absence or presence of Nb3 or Nb4 is depicted. Black dots represent binding sites for Nb3 and Nb4 in hTNC. **(B)** Quantification of Nb3 and Nb4 binding according to the position (nm) on TNC Representation of a TNC monomer with oligomerization domain (triangle) to form hexamers as seen in **(A)**, FNIII repeats (gray boxes, constant domains, white boxes, alternative domains) and fibrinogen like domain (circle). Representative result of three independent experiments, displaying the quantification of 500 micrographs.

We investigated the location of the antigenic epitope in hTNC by negative electron microscopy where the nanobodies were coupled to gold beads. Upon incubation of hTNC with gold beads-bound nanobodies, we detected the gold particles along the length of the hTNC monomers and quantified them (Figure 2A). We observed a high number of Nb3 and Nb4 binding in the middle of the hTNC monomer resembling binding of several soluble factors in the fifth fibronectin type III repeat (FNIII) in TNC [TN5 (64)]. Nb3 and Nb4 showed a similar binding pattern suggesting that both nanobodies recognize the same or overlapping epitopes in hTNC, presumably TN5 (Figure 2B).

### Nanobodies Nb3 and Nb4 Recognize hTNC in Fresh Frozen and Paraffin-Embedded Human Tissues

**Figure 3:**
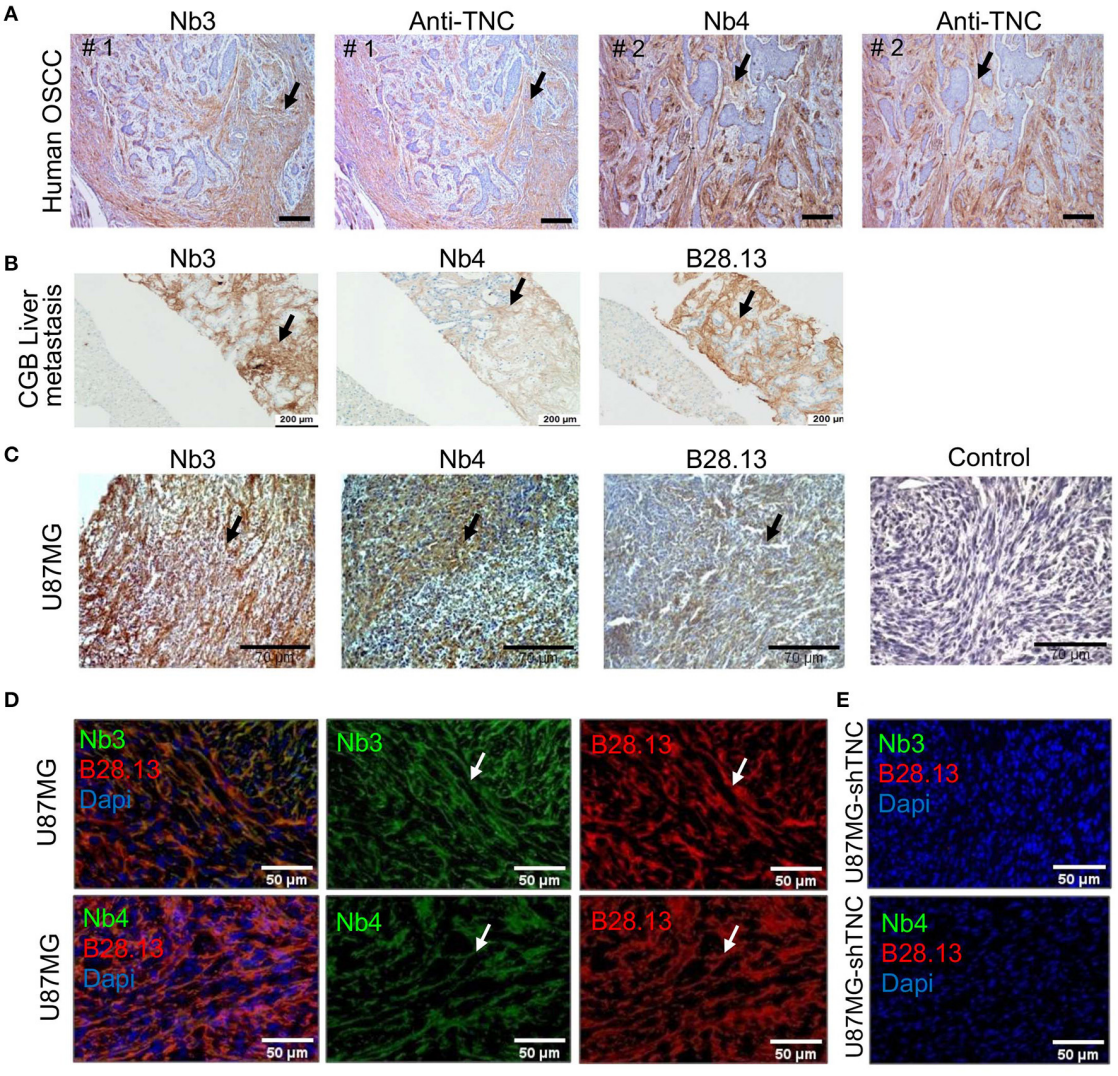
Detection of TNC in tissues by Nb3 and Nb4 IHC **(A–C)** and IF analysis **(D,E)** with Nb3 and Nb4 **(A–E)**, B28-13 **(B–E)** and a polyclonal anti-TNC antibody (Anti-TNC) **(A)**. **(A)** Human OSCC (FFPE), **(B)** liver metastasis from a gall bladder carcinoma (FFPE), **(C–E)** U87MG tumors, **(C)** FFPE, **(D,E)** PFA fixed tissue. Scale bar, 100 μm **(A)**, 200 μm **(B)**, 70 μm **(C)** and 50 μm **(D,E)**.

Next, we investigated whether Nb3 and Nb4 recognized hTNC in FFPA tissues. Therefore, we stained human colon tissue from an ulcerative colitis (UC) patient and noticed a staining pattern that resembled published TNC expression in this tissue (65–67), (Supplementary Figure 3A). We also stained tissue from human tongue tumors (OSCC) with Nb3 and Nb4 and with a commercial rabbit polyclonal anti-TNC antibody on an adjacent section, and observed similar staining patterns for TNC reminiscent of tumor matrix tracks (TMT) that have previously been described [Figure 3A, (22)]. We also stained a liver metastasis from a patient with a carcinoma of the gall bladder (CGB) (Supplementary Table 1) with Nb3 and Nb4 and observed a strong immunoreactivity of the stroma, similar to the staining observed with the anti-TNC antibody B28.13 (Figure 3B). This stromal staining resembled that of TNC expression in biliary tract cancers, including CGB liver metastasis as seen by conventional IHC (68).

Next we addressed recognition of murine and human TNC in tissues by IHC and IF. Therefore, we stained U87MG glioblastoma xenografted tumors where it was previously noticed that human TNC was largely more abundant than murine TNC by IHC and IF (56). We observed a fibrillar TNC signal in the U87MG tumors by IHC with Nb3 and Nb4 (Figure 3C) that overlapped with that of the B28.13 antibody signal, confirming specificity of the nanobodies for TNC (Figure 3D). As the U87MG tumors also express murine TNC but at much lower abundance (56), we stained U87MG tumors with a knockdown for human TNC in the grafted tumor cells and did not see a signal, suggesting that Nb3 and Nb4 at the chosen dilution recognize predominantly human TNC (Figure 3E).

### Nanobody Nb4 Counteracts the Anti-adhesive Properties of TNC on a FN/TNC Substratum

**Figure 4:**
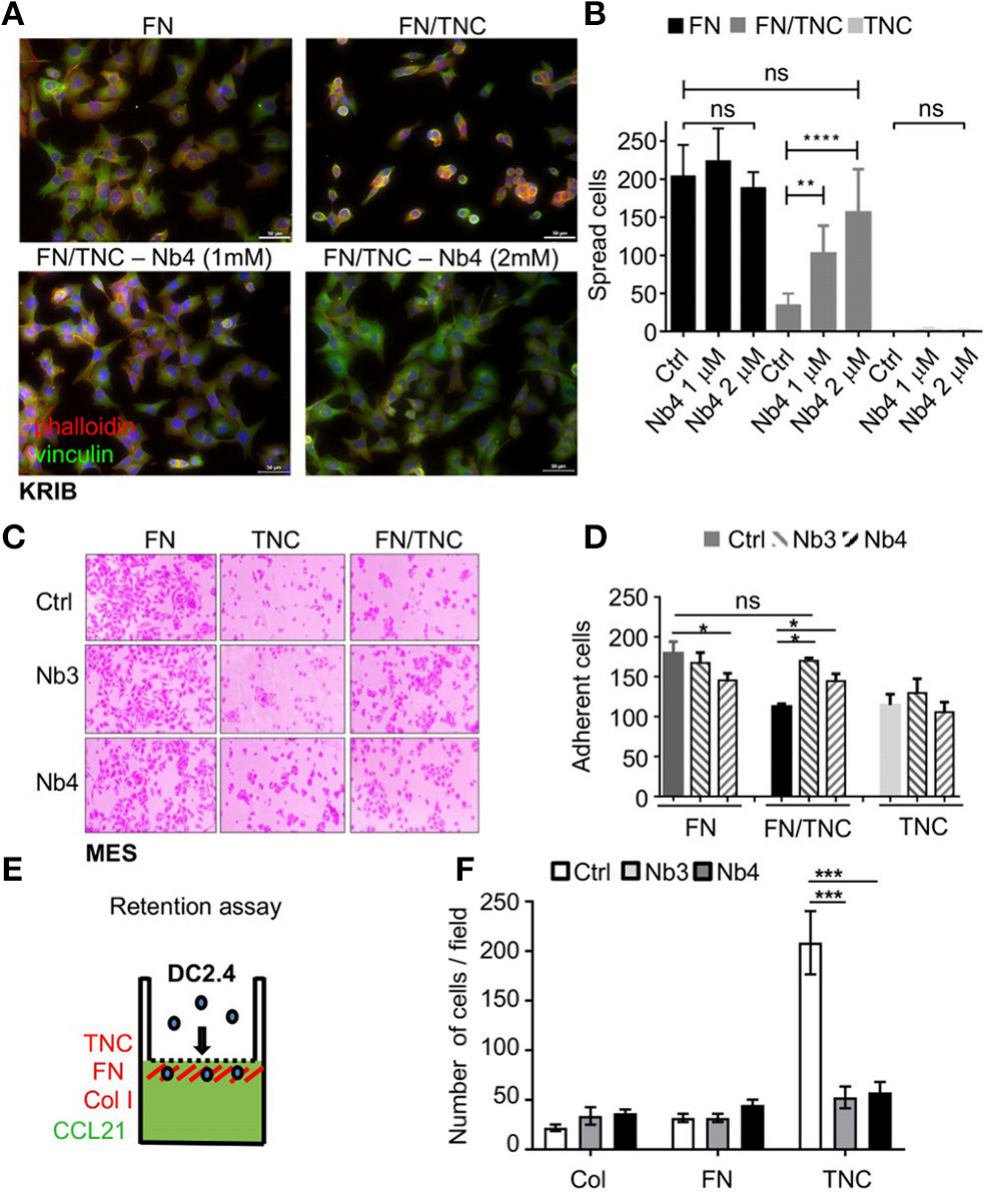
Nb3 and Nb4 interfere with TNC functions. **(A,B)** Cell spreading assay followed by quantification of KRIB cells plated on FN, a mixture of FN and hTNC (top panels) or a mixture of FN and hTNC incubated with Nb4 (bottom panels). After 2 h, the KRIB cells were fixed and stained with phalloidin (red) to detect polymerized actin, or an anti-vinculin antibody (green) to detect focal adhesion complexes and the nuclear marker DAPI (blue). Higher magnifications are shown in Supplementary Figure 4. Spread cells were counted **(B)**. *N* = 3 experiments, *n* = 2 wells. **p < 0.01, ***p < 0.001. Two-way ANOVA test. **(C,D)** Adhesion of mesangial cells (MES) 2 h after plating on FN, TNC or FN/TNC without (Ctrl) or with Nb3 and Nb4, respectively followed by quantification **(D)**. *N* = 3 experiments, *n* = 2 wells. *p < 0.05. Two-way ANOVA test. **(E)** Schematic representation of the Boyden chamber transwell chemoretention assay with DC2.4 toward CCL21 in the bottom well. The lower surface of the insert was coated with FN, Col I, or hTNC, respectively. **(F)** Quantification of DC2.4 cells on the coated surfaces upon migration toward CCL21 (5 h after plating) and pretreatment or not (Ctrl) with Nb3 and Nb4, respectively. Note that Nb3 and Nb4 significantly abolished DC2.4 cell retention by TNC/CCL21. *N* = 2 experiments, *n* = 4 wells. Mean ± SEM, Kruskal-Wallis test and Dunn's post-test. ***p < 0.005, ****p < 0.0001.

By using human osteosarcoma KRIB cells, we investigated whether Nb4 had an impact on cell rounding by TNC on a FN/TNC substratum. Previously, we had shown that cells are inhibited by TNC to spread on a combined FN/TNC substratum since TNC competed syndecan-4 binding to FN (44, 69). Here, we plated KRIB cells on FN, FN/TNC and TNC, respectively with or without Nb4. By staining with pholloidin (polymerized actin) and anti-vinculin (focal adhesions), we confirmed cell spreading on FN and cell rounding on FN/TNC and TNC, respectively. While cells had some actin stress fibers and focal adhesions on FN, addition of Nb4 did not change that (Figures 4A,B, Supplementary Figure 4). However, upon addition of Nb4 to cells plated on FN/TNC we observed that cells spread in a Nb4 dose dependent manner with actin stress fibers and focal adhesions that looked similar to those in cells plated on FN (Figures 4A,B, Supplementary Figure 4). As in KRIB cells, both Nb3 and Nb4 restored adhesion of mesangial cells (MES) on a FN/TNC substratum suggesting that Nb3 and Nb4 blocked binding of TNC to FN (Figures 4C,D).

### Nanobodies Nb3 and Nb4 Abolished DC2.4 Chemoretention by TNC/CCL21

Previously, we had shown that in combination with CCL21 TNC immobilized dendritic DC2.4 cells (22). Here we used a Boyden chamber transwell migration assay to investigate whether Nb3 and Nb4 impacted chemoretention by TNC. Therefore, we coated the lower surface of the insert with FN, collagen I (ColI) or TNC and added Nb3 or Nb4, respectively, and measured DC2.4 cell migration toward CCL21 placed in the lower chamber. We measured cells adhering on the coated surfaces in the presence or absence of the nanobodies and observed first a high number of cells being tethered on TNC but not the other coatings. Second, we noticed that the number of adherent cells dropped on TNC to that of the other coatings upon addition of Nb3 and Nb4 whereas no difference was seen with the other matrix coatings (Figures 4E,F).

### Three Dimensional Topology of the Nb3/TN5 Interaction Complex

**Figure 5:**
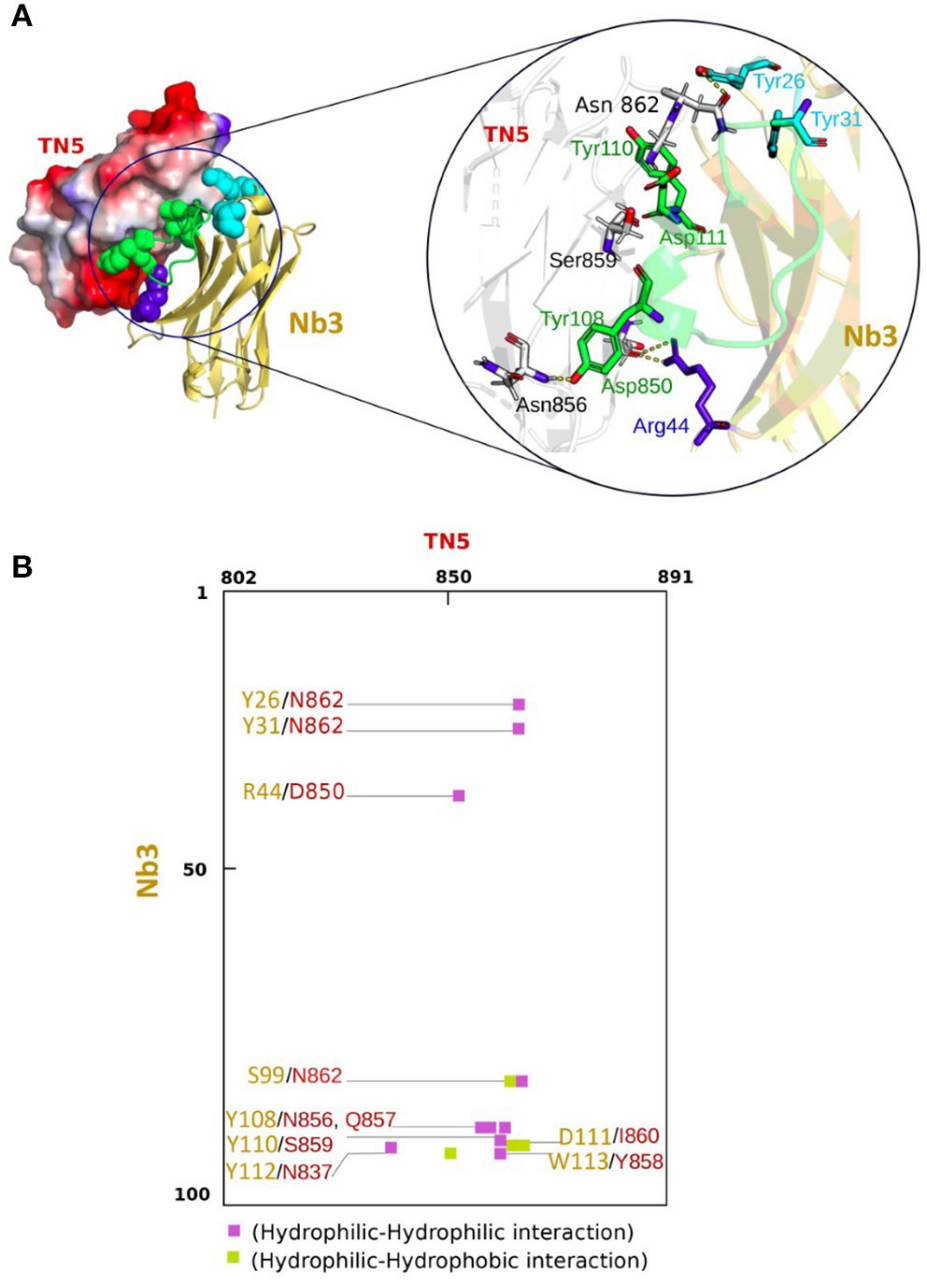
Modeling of the three dimensional topology of the Nb3/TN5 interaction complex and information about amino acid contacts. **(A)** Model of the Nb3/TN5 complex TN5 (red) and Nb3 (yellow) are shown with contacting amino acids in blue and green (left). Magnification (circle) represents a spatial view on identified amino acid residues (three letter code, position in TN5 and Nb3 indicated by a number, respectively) generating relevant hydrophobic, electrostatic and H-bond interactions (right). TN5 is represented as white surface with electrostatic surface coloring. Nb3 is presented in transparent yellow. The most implicated amino acid residues forming H-bonds are labeled with a dashed yellow line. **(B)** Contact interaction map of TN5 with Nb3 representing amino acids 802–891 in TNC (top) and amino acids 1–100 in Nb3 (left). Violet (hydrophilic-hydrophilic), green (hydrophobic-hydrophobic) and yellow (hydrophilic-hydropholic) boxes represent the properties of the interaction.

We adopted a computational structure analysis strategy in order to identify amino acid residues involved in the interaction of Nb3 with TN5. The 1f2x (chain L), 3l95 (chain B), 5ocl (chain G) and 5vak (chain B) structures were used as principal template for Nb3-frameworks, CDR1, CDR2 and CDR3, respectively. The number of generated Nb3 models was set to 1,000. The top ten model best scores, ranked according to an energy-based scoring were selected from 10 clusters composed of 100 structures per cluster then visually double checked (to detect structural anomalies) using the molecular visualization PyMOL software. The TN5 structure (1TEN) was included in the Nb3 molecular docking simulation. The positively and negatively charged residues are in blue and red, respectively, whereas the neutral side-chains are indicated in white, showing clearly separated charges on the TN5 surface. Using the docking approach, we generated the top ten possible binding sites, ranked according to an energy-based scoring and filtered them to get an unique complex presenting the best molecular orientation with the most stable position (Figure 5A). In order to assess the main amino acid residues involved in the molecular Nb3-TN5 interaction, we used the COCOMAPS (bioCOmplexes COntact MAPS) web application server. A predicted intermolecular contact map of the Nb3-TN5 complex is illustrated in Figure 5B with a cut-off of 3 Å, highlighting the most crucial residues mainly implicated in the complex interaction. The predicted binding site residues in TN5 are as follow: N862, D850, N856-Q857, S859, I860, N837, Y858 interacting with Nb3 at position Y26, Y31, R44, S99, Y108, Y110, D111, Y112, W113. Interestingly, the CDR2 and CDR3 are predicted to dominate the interaction with TN5. Details of crucial residues involved in H bounds as proton donors or acceptors are described in Supplementary Table 3.

## Discussion

The matrix, a highly abundant component of tumors, could be considered as a good tumor biomarker as matrix is often more stable than e.g., antigens expressed by tumor cells (70, 71). Furthermore, matrix seems to be accessible to antibodies and antibody derivatives in therapy (33). Detection of abundant tumor specific matrix could be useful for monitoring tumors and their progression. In this context, TNC is an intriguing matrix molecule, as it is highly expressed not only in tumors, but also in fibrosis and chronic inflammation often correlating with disease progression (4, 11, 72, 73). Hence, detection of high TNC levels in tissues and body fluids of patients with e.g., cancer or rheumatoid arthritis is a promising strategy (21, 22, 62). The large isoform of TNC, highly abundant in cancer tissue, may even be a good address for the delivery of drugs into the tumor (1). In particular, TNC-specific antibodies were shown to be a means for tumor targeting. In the past, several TNC-specific monoclonal antibodies were developed as well as aptamers and antibody fragments (scFv) that are currently undergoing clinical evaluation (37, 74–78). The anti-human TNC G11 antibody (79) was used for targeting TNC in glioma xenografts upon coupling with ^18^F-fluorodeoxyglucose (37). Phase I and II clinical trials were performed with the F16 anti-TNC antibody in glioma patients (80, 81), breast cancer (82, 83) and Hodgkin's lymphoma (35). Coupling the TNC specific F16 antibody to IL-2 (Teleukin®) was used to deliver IL-2 into the cancer tissue (82, 84). Moreover, since 2013, a phase II clinical trial using Teleukin® labeled with ^131^Iodine is in progress in melanoma patients (EudraCT 2012-004018-33) (35). These antibodies did not show any adverse effects and may be useful for tumor imaging. One needs to await the outcome of the clinical studies to see whether targeting TNC with these antibodies can also reduce tumor growth and potentially tumor progression (78).

Staining of FFPE tissues remains a challenge in the clinical practice due to frequent masking of epitopes. Also access of antibodies to their epitopes used in functionalized antibody assisted drug delivery remains a challenge. Due to their intrinsic characteristics, such as small size, high stability and good specificity, nanobodies constitute promising agents to overcome some of these limitations (85). Indeed, in a recently patented study covering another group of TNC specific nanobodies (86, 87), the authors demonstrated that the radioactive coupled nanobody ^64^Cu-NJTs detected micrometastasis in tumor mice by life imaging. These results promise that TNC specific nanobodies could be used for delivery of therapeutic compounds into tumors or into other tissues with high TNC content.

In this report we aimed at the development of nanobodies directed against hTNC to detect TNC in FFPE tissues and to block TNC functions. We purified recombinant TNC of human origin and used this molecule to elicit a potent immune response in the dromedary. A substantial proportion of polyclonal heavy chain IgG subclasses bound to TNC and recognized TNC by IF staining in tumor tissue (51). This encouraged us to generate nanobodies. A VHH library from this dromedary was generated that met the required quality control standards (88, 89) and allowed us to isolate nanobodies that specifically recognized TNC. Although, the titer of dromedary antibodies against hTNC was significantly high, the *in vitro* selection of anti-hTNC binders from this specific VHH library allowed us to retrieve only eight binders after three rounds of bio-panning. A possible explanation of this limited sequence diversity of binders might be attributed to our screening condition as we immobilized hTNC which potentially has masked the epitopes or prevented nanobodies to bind due to sterical hindrance. To retrieve nanobodies recognizing additional TNC sequences, future biopanning could be done by using soluble TNC.

In this paper, we have generated eight human TNC specific nanobodies and the two best in class candidates (Nb3, Nb4) in terms of binding strength and specificity were further characterized in more detail. The amino acid sequences of the three TNC-specific Nb revealed a high degree of identity with human VH sequences of family III; however the VHH imprints were clearly present (90, 91).

Nb3 and Nb4 recognized specifically TNC by immunoblotting, ELISA and negative EM imaging and by staining of PFA and FFPE fixed human tissues. We identified binding of Nb3 and Nb4 in the center of the TNC monomer around TN5 which may be a particularly exposed site in TNC as TN5 was shown to bind several soluble factors (22, 64). Future studies have to address whether TN5 is particular in raising an immune response. It is interesting to note N-glycosylation sites in TN5 and that N-glycosylation is important for the envelope proteins of HIV to bind TNC (10). In this context it will be interesting to learn more about the antigenic epitope properties of the NJTs nanobodies and to see whether the epitopes are different.

Both nanobodies exhibited a TNC specific staining in all tested tissues confirming the aptitude of Nb3 and Nb4 to recognize native TNC *in situ*. As our study is limited to a few examples as proof of concept, more stainings of FFPE embedded tissues have to be done in the future. It is important to compare Nb3 and Nb4 stainings with established anti-TNC antibody staining protocols to determine whether staining patterns are similar or different. *In silico*, 3D modeling of the interaction of Nb3 with TN5 revealed the potential contribution of CDR2 and CDR3 in the interaction with critical hydrophobic amino acid residues in TN5. Future site directed mutagenesis experiments have to evaluate the predicted nanobody-TN5 interaction sites in particular taking into account a potential role of N-glycosylation.

The Nb3 and Nb4 nanobodies may be suitable for a sandwich ELISA assay. There is a need for a robust ELISA assay to detect TNC in body fluids such as blood and urine to be used as parameters for earlier diagnosis of diseases with high TNC levels (11, 12, 92, 93). Only few commercial ELISA kits are available. A frequently used one recognizes the FNIIIB domain that is not present in all TNC proteoforms and thus may miss TNC species lacking this domain (94). Thus, an advantage of using Nb3 and Nb4 for ELISA is that they recognize an epitope in the constant FNIII domains (likely in TN5) thereby potentially detecting more TNC isoforms.

To respond to the need of high Nb3 and Nb4 yields for applications mentioned above the expression conditions have to be optimized in the future as the production yields of Nb3 and Nb4 varied and were not very high.

Finally, we observed that Nb3 and Nb4 recognized not only human TNC but also murine TNC as seen by immunoblotting and IFT with a K_D_ value in the three digits nanomolar range comparable to other molecules binding TNC [TGF-β1, K_D_ = 20.3 nM (64); CCL21, K_D_ = 58 nM (22); FN III13, K_D_ =128 nM (44)]. Therefore, these nanobodies may be useful for preclinical models assessing tumor growth by life imaging, delivery of drugs into tissues with high TNC levels or even to inhibit TNC actions in tumors as we observed that both Nb3 and Nb4 inhibited TNC-induced cell rounding, and TNC specific retention of immune cells in the matrix. Thus, our nanobodies could be suitable to inhibit TNC functions in cancer cell migration and invasion and to ablate immune-suppressive functions of TNC in cancer. As a major binding site for the envelope protein in HIV was found in TN5 (10), Nb3 and Nb4 may be useful to modulate this interaction. Finally, Nb3 and Nb4 may also be useful to target TNC actions in COVID19 as high TNC levels correlated with severity of the disease symptoms (13). Our results provide a rationale for a future clinical evaluation of the hTNC-specific Nbs.

## Data Availability Statement

The raw data supporting the conclusions of this article will be made available by the authors, without undue reservation.

## Ethics Statement

The Institutional Review Board of the Centre de Ressources Biologiques (Association française de normalization: 2010/39043.2) of the Hautepierre hospital (Strasbourg, France) and Centre Paul Strauss have approved the study on human Ulcerative Colitis samples and human OSCC, respectively. The patients/participants provided their written informed consent to participate in the study.

## Author Contributions

SD contributed by library construction, nanobody selection, characterization of the nanobodies, and writing the manuscript. RB contributed to the library construction and nanobody selection. WE, TL, CA-F, and AKs contributed by characterization of the TNC blocking functions of the nanobodies. DM, AKs, SB, and IH contributed to the characterization of the nanobodies. MM contributed to characterization of the nanobody TNC interaction by negative electron microscopy. ZB contributed to the dromedary veterinary management and immunizations. RC-E contributed by funding, supervision, and validation. GO contributed by funding, supervision, validation, manuscript writing, and review and editing. BB-Z led the project, contributed by funding, supervision, validation, manuscript writing, and review and editing. All authors contributed to the article and approved the submitted version.

## Conflict of Interest

MM was employed by Colzyx AB. The remaining authors declare that the research was conducted in the absence of any commercial or financial relationships that could be construed as a potential conflict of interest.

## Footnotes

^1^The Rosetta software, https://www.rosettacommons.org/software/license-and-download
