# Supplementary material for: Novel Human Tenascin-C Function-Blocking Camel Single Domain Nanobodies
Source: Front Immunol. 2021 Mar 15;12:635166. doi: 10.3389/fimmu.2021.635166 (PMC8006918; doi:10.3389/fimmu.2021.635166)
Supplement: Supplementary file 1 [file Data_Sheet_1.docx]

**Supplemental information for Dhaouadi et al., “Novel human tenascin-C function blocking camel single domain nanobodies”**

**Supplemental Figures S1 – S4**

**Supplemental Tables S1 – S3**

**Supplemental Figure S1**

| **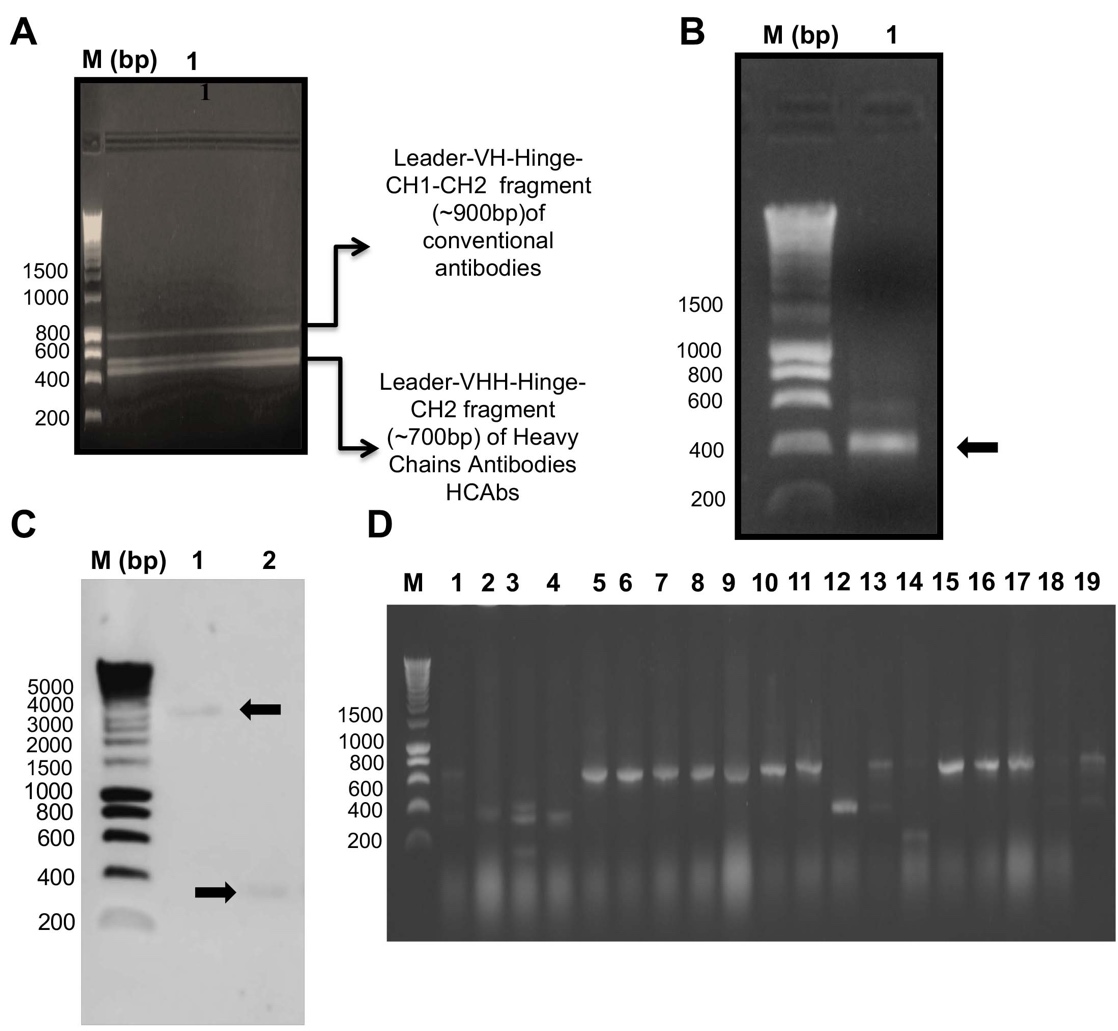** |
| --- |
| **** |

**Supplemental Figure S1** **Isolation of hTNC specific nanobodies** VHHs genes were generated by PCRs. Final PCR products were sequentially digested with NcoI and NotI and cloned into the digested phagemid vector pMECS.

**(A)** Analysis of PCR products from the dromedary antibody fragments (using the primers CALL001 and CALL002) by electrophoresis in a 1% agarose. The upper band of 900 bp corresponds to DNA fragments (VH-CH1-hinge region-part of CH2) of conventional IgG1, whereas the lower band of 700 bp corresponds to DNA fragments (VHH-hinge-region-part of CH2) of non-conventional IgG2 and IgG3 isotypes. M: Molecular weight marker, Lane 1: PCR products with indicated size.

**(B)** Analysis of nested PCR products obtained by reampliﬁcation of the 700 bp fragment, using sense primer SM017 and the antisense primer PMCF by electrophoresis in a 1% agarose gel. The band of 400 bp corresponds to VHH fragments (V–D–J-REGION of non conventional IgG2 and IgG3 antibodies). M: Molecular Weight Marker, Lane 1: Nested PCR Product.

**(C)** Analysis of digested pMECS phagemid and VHH inserts by gel electrophoresis in a 1% agarose gel. M: Molecular Weight Marker Lane 1: pMECS phagemid double-digested with NcoI, NotI and XbaI. Lane 2: Nested PCR Product (VHH) double-digested with NcoI and NotI.

**(D)** To assess percentage of clones with proper insert of the cloned VHH, 19 single colonies of the transformants were randomly picked and their DNA was used for colony PCR with MP57 and GIII primers. The amplicons were separated on a 1% agarose gel. Clones without VHH DNA insert have a smaller amplicon size. Note, that 78.94% of clones have an insert of the correct size of 700 bp.

**(E)** ELISA on periplasmic extracts. The periplasmic extract from 25 randomly picked clones derived from the third bio-panning round was analysed with ELISA. hTNC at 1 µg/ml was coated in each well. Periplasmic exctracts obtained from clones selected against the scorpion *Androctonus Australis Hector* (AahI, IC (1)) and *Buthus occitanus tunetanus* (BotI, IC (2)) toxins were used as irrelevant controls (IC). A total of eight clones against hTNC were selected on the basis of absorbance. The x-axis shows the clone number and the y-axis the absorbance at 492 nm.

**Supplemental Figure S2**

**Supplemental Figure S2 Binding specificity of Nb3 and Nb4 towards hTNC**

One hundred microliters of hTNC (0.5 μg / mL) were coated onto microtiter plates, and 100 μL nanobodies (5 μg / ml) were added. After incubation with mouse anti-HA tag antibody and then anti-mouse HRP, absorbance at 492 nm was measured by an ELISA reader. Anti-BotI nanobody (5 μg / ml) was used as irrelevant control (IC). Error bars indicate the standard deviation of the mean values resulting from triplicates.

**Supplemental Figure S3**

| **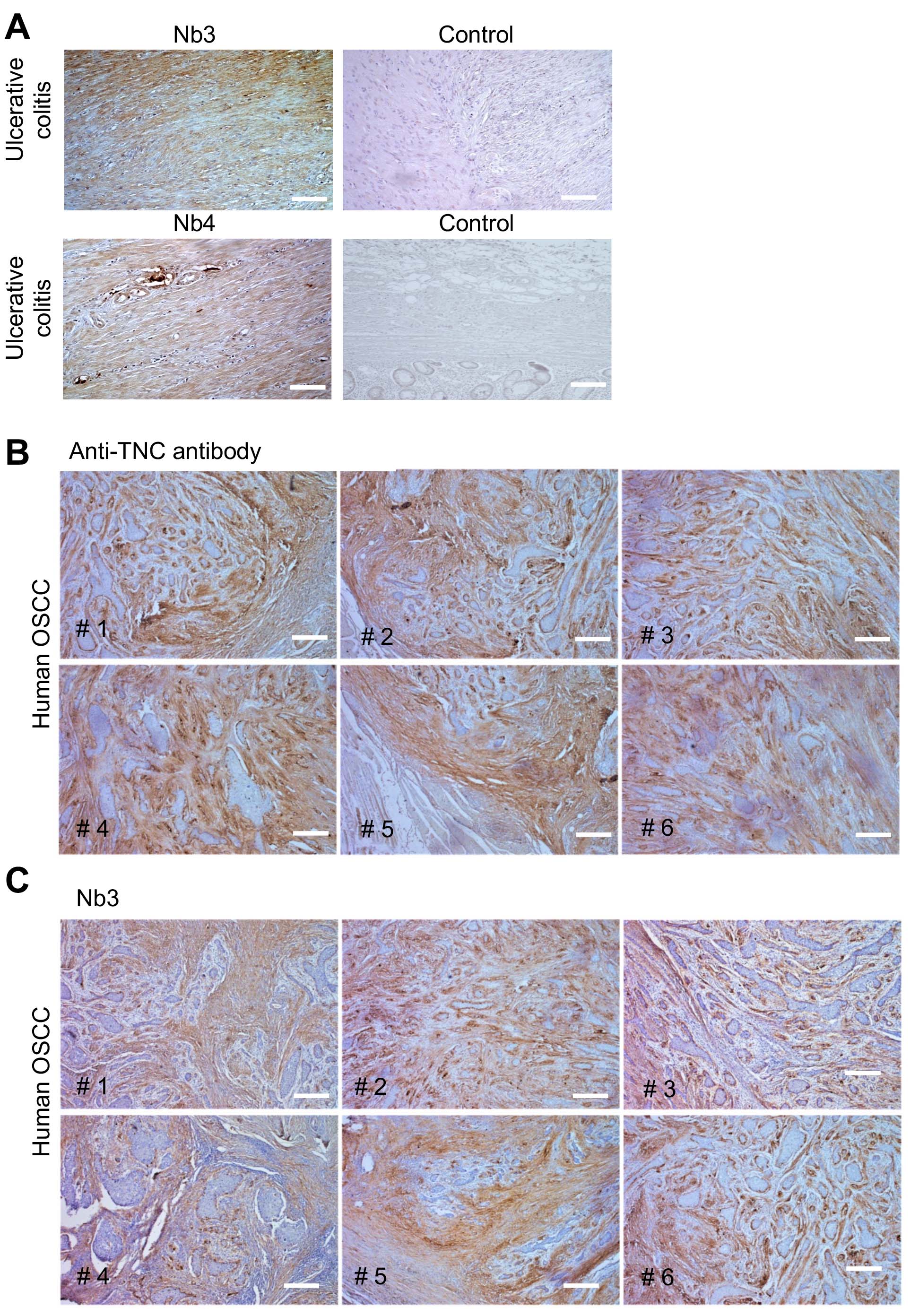** |
| --- |
| **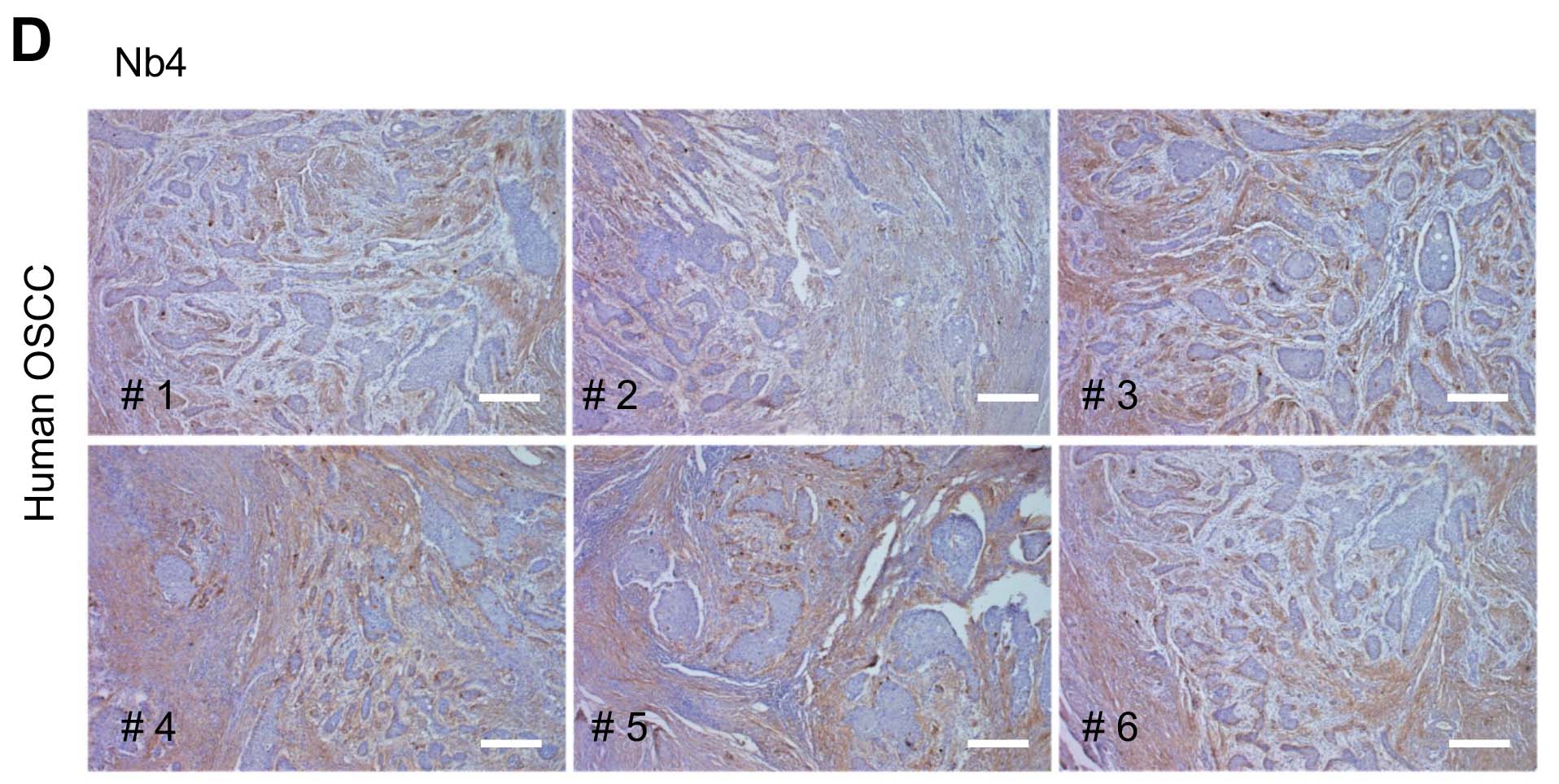** |

**Supplemental Figure S3 Detection of TNC in human tissues with hTNC-specific Nb3 and Nb4**

**(A)** Representative IHC staining images for TNC in FFPE embedded tissues from human ulcerative colitis. Staining of TNC with Nb3 and Nb4. Control, no anti-HA secondary antibody. Scale bar, 70 µm.

**(B - D)** Representative IHC staining images for TNC in FFPE embedded tissues from human tongue OSCC. Detection of TNC with a rabbit polyclonal anti-TNC antibody (**B**), Nb3 (**C**) or Nb4 (**D**). Scale bar, 100 μm.

**Supplemental Figure S4**

**Supplemental Figure S4**

KRIB cells were plated on surfaces coated with FN or a mixture of FN and hTNC plus Nb4. After 2 hours, cells were fixed with PFA and stained with phalloidin (red) to reveal polymerized actin, and an anti-vinculin antibody (green) to detect focal adhesions, and the nuclear marker DAPI (blue). Scale bar, 20 μm.

**Supplemental Tables**

**Supplemental Table S1 Characteristics of tumor patients**

| **Sample ID** | **Tumor location** | **Sex** | **Stage** | **Metastasis** | **Sampling date** |
| --- | --- | --- | --- | --- | --- |
| 1 | Tongue base | F | T4N0-1M0 | no | 1989 |
| 2 | Mobile tongue | M | T4N0-1M0 | yes | 1991 |
| 3 | Tongue base | M | T4N0-1M0 | no | 1992 |
| 4 | Tongue base | F | T4N0-1M0 | yes | 1992 |
| 5 | Mobile tongue | M | T4N0-1M0 | yes | 1994 |
| 6 | Tongue base | M | T4N0-1M0 | yes | 1995 |
| 189 | Liver Metastasis from CGB | F | T3N2M1 | yes | 2015 |

Human cancer tissues from oral squamous cell carcinoma (OSCC) with annotation of OSCC tumor location and liver metastasis derived from gall bladder carcinoma (CGB). Gender (F, female, M, male), staging (Tumor Node Metastasis (TNM) classification) are shown.

**Supplemental Table S2:** Enrichment of hTNC-specific phages during subsequent rounds of panning

| Round of panning | Input phages (pfu/ml) | Output phages from hTNC coated-wells |
| --- | --- | --- |
| Round 1 | 5.4 × 10^10^ | 1.3 × 10^8^ |
| Round 2 | 1.0 × 10^11^ | 1.7 × 10^9^ |
| Round 3 | 1.0 × 10^11^ | 1.0 x 10^9^ |

**Supplemental Table S3** Amino acid residues generating H-bond interactions between Nb3 and TN5 with donor and acceptor atoms details.

| **Donor** | | | | **Acceptor** | | | | **Dist (Å)** | |
| --- | --- | --- | --- | --- | --- | --- | --- | --- | --- |
| Res1 | N° res1 | Atom1 | Chain1 | Res2 | N° res2 | Atom2 | Chain2 | Dist H-Bonds | Dist CA-CA |
| ARG | 44 | NH2 | H | ASP | 850 | O | A | 2.17 | 10.2 |
| ASN | 856 | N | A | TYR | 108 | OH | H | 1.87 | 8.54 |
| SER | 859 | N | A | TYR | 108 | O | H | 2.6 | 5.2 |
| SER | 859 | OG | A | ASP | 111 | OD1 | H | 3.26 | 4.69 |
| TYR | 112 | OH | H | GLY | 861 | O | A | 2.86 | 6.56 |

Residue (Res), Dist (Distance), Angstrom (Å),Carbon Atom (CA).
